# New Methods in Exploring Old Topics: Case Studying Brittle Diabetes in the Family Context

**DOI:** 10.1155/2016/6437452

**Published:** 2015-11-08

**Authors:** Moritz Philipp Günther, Peter Winker, Stefan A. Wudy, Burkhard Brosig

**Affiliations:** ^1^Psychoanalytic Family Therapy, Center of Child and Adolescent Medicine, Justus-Liebig-University, Feulgenstraße 10-12, 35392 Giessen, Germany; ^2^Department of Economics, Justus-Liebig-University Giessen, Licher Straße 64, 35394 Giessen, Germany; ^3^Pediatric Endocrinology & Diabetology, Center of Child and Adolescent Medicine, Justus-Liebig-University, Feulgenstraße 10-12, 35392 Giessen, Germany

## Abstract

*Background*. In questing for a more refined quantitative research approach, we revisited vector autoregressive (VAR) modeling for the analysis of time series data in the context of the so far poorly explored concept of family dynamics surrounding instable diabetes type 1 (or brittle diabetes). *Method*. We adopted a new approach to VAR analysis from econometrics referred to as the optimized multivariate lag selection process and applied it to a set of raw data previously analyzed through standard approaches. *Results*. We illustrated recurring psychosomatic circles of cause and effect relationships between emotional and somatic parameters surrounding glycemic control of the child's diabetes and the affective states of all family members. *Conclusion*. The optimized multivariate lag selection process allowed for more specific, dynamic, and statistically reliable results (increasing *R* ^2^ tenfold in explaining glycemic variability), which were derived from a larger window of past explanatory variables (lags). Such highly quantitative versus historic more qualitative approaches to case study analysis of psychosomatics surrounding diabetes in adolescents were reflected critically.

## 1. Introduction

Sigmund Freud is rarely mentioned in scientific discourse without also belittling the lack of quantitative statistical evidence for his elaborate models. At the same time, his qualitative case reports and the conclusions he drew from them by far belong to the most well-known research in psychosomatic medicine. Despite all valid critique, one reason, we argue, may very well be the superiority of the single case study in first observing, describing, capturing, evaluating, and creatively reflecting on an infinite set of parameters surrounding any chosen topic. Out of this primary assessment, novel hypotheses and further (more costly) research may emerge.

It is our objective to reapply such primary assessment to the case of adolescent brittle diabetes (or more generally speaking, the psychosomatic underpinnings of diabetes type 1 in minors and young adults), while also trying to answer calls for more quantitative and statistically reliable approaches to doing so. This in mind, we have first selected a highly quantitative case study on family dynamics and brittle diabetes [1] and reviewed and reanalyzed its raw data through implementation of a new statistical procedure increasing the coefficient of determination in the new model by factor ten (while also presenting new and clearer findings), in order to then, in a second step, discuss and compare our results to possibly the historically most well-known set of qualitative case studies on the topic [2].

We will start by briefly revisiting the literature on the psychosomatics of adolescent instable diabetes type 1, present a case vignette and basic data collection method of the original case study we reexamine (which may be skipped by those familiar with the work published by [1]), followed by a detailed description of our new statistical approach and its results, concluding with a clear clinically oriented graphical presentation of our findings and their discussion in light of Minuchin et al.'s [2] qualitative findings.

*The Case of “Brittle” Diabetes*. One out of 600 US or European school-age children suffers from insulin dependent diabetes mellitus [3, 4]. Just about 33 percent of diabetics between 13 and 19 years of age manage to maintain tolerable glycemic control and a HbA_1c_ below 8; 6.3 percent suffered at least one episode of major hypoglycemia within the last three months [5, 6]. The devastating immediate and long-term effects of poor diabetic control are widely known and feared. 44 percent of the variance in blood glucose control can be statistically explained by psychological variables in these patients and their parents [7]. A randomized controlled study further demonstrates how an intensive inpatient treatment program including psychoanalytic psychotherapy could effectively improve diabetic control in children [8]. These cases of glycemic instability with no somatic explanation have been termed “brittle diabetes” by some authors [9] and there is no doubt concerning the importance of further exploration of the causes and remedies surrounding this truly psychosomatic disease.

While various aspects of brittle diabetes have been explored in recent years, including its exact definition, there seems to be a gap in the literature in exploring how emotional variables of all individuals within the family system may interact to affect glycemic control of the diabetic adolescent, the “index patient” of a dysfunctional family system. The little research which has sought to fill this gap (i.e., [2, 10, 11]) is primarily qualitative in nature and must face similar critique as all such work, as will be discussed in the last section of this study.

*The Case and Its Psychosomatic Background (adopted and revised from [1])*. The adolescent index patient of this case study was diagnosed with diabetes type 1 at age of four (clinical clues were polyuria, polydipsia, loss of appetite, a fungal infection, HbA_1c_ of 9.1 per cent, antibodies against islet cells, and GAD65).

Family dynamics surrounding this classic family of three (biological parents, single child) appeared unsuspicious notwithstanding the girl's history of poorly controlled bronchial asthma and allergic diseases.

Yet at age of six, nocturnal hypoglycemia with loss of consciousness led to readmission to the hospital, during which another episode of profound hypoglycemia, this time in conjunction with a tonic-clonic seizure, occurred, thus further consolidating her parents' distress concerning hypoglycemia and hospital treatment. Once all educational efforts concerning the diabetic management were exhausted (including individual and family-based counseling, detailed and repetitive disease-specific education, and information about glycemic control mechanisms including the influence of nutrition, sport, and other aspects of blood sugar regulation), but a HbA_1c_ below 7 percent was never achieved, the family finally sought for psychosomatic family treatment. Psychodynamically based therapeutic analysis of the family dynamic suggested a conflict between the adolescent and her mother about who had control of the blood sugar levels. The mother's dominance seemed to have negative effects on her daughter's glycemic control. Fears of hypoglycemia were somewhat irrational with all three family members, including the father, who, at first sight, seemed rather more distant to the matter (literature proposes parental hypoglycemia avoidance behaviours to adversely affect glycemic control [12]).

Six family therapy sessions were undertaken on a biweekly schedule. The family's shock in relation to the diagnosis and mistrust of hospital personnel was discussed.

Finally, a therapeutic intervention confronted them with their specific type of collusion concerning (in-)dependence, in which both parents, in their manifest statements, advocated for more self-confidence and extended duties on the side of the daughter, but on a more latent level, gave hints to their “beloved little girl” not being ready to take control over the blood sugar monitoring by herself. This mostly unconscious conflict had culminated in cloudy paths of communication concerning glycemic control, in nebulous distributions of duties within the family members, and, as a result of the arrangement, in deep dissatisfaction over the failure of proper diabetic control.

## 2. Methods

### 2.1. Collecting Quantitative Data

While traditional case studies would focus on the qualitative data outlined above, we sought to amend such observations by a highly quantitative approach in order to produce more evidence based and reproducible results. Therefore, we aimed to statistically explore how specific basic affect states of all three individual family members may impact each other and the success of the diabetic management over a period of 120 days. To operationalize this quest, we drew on the standardized self-assessment manikin (SAM), as developed by Bradley and Lang (for details see [13, 14]), asking all three family members to individually record on a daily basis their valence (mood), arousal (high versus low), and dominance (a sense of presence in the current environment). In addition the index patient was asked to obtain at least three daily blood glucose measurements (or more if required by the disease) over the same period utilizing a common standardized technique. This form of diary based data collection is also referred to as ecological momentary assessment with many benefits in terms of accuracy and validity of measurements [15].

Standard deviations of the daily blood glucose measurements served as an indicator for glycemic variability, a measure which recent research has identified as the most precise predictor of diabetic control, followed by the HbA_1c_-value in second place [16–19], due to it being the best known predictor for diabetic complications and microvascular derailments in particular [20].

Resulting from this data collection and primary analysis are ten time series: three time series for each of the three family members from the SAM, affective valence (happy, sad), arousal (excited, calm), and dominance (a sense of presence, distance to the current environment), as well as one time series recording glycemic variability (daily standard deviations of measurements). In contrast to Günther et al. [1], these ten time series were further analyzed by a completely new statistical approach to vector autoregressive (VAR) modeling. While past analysis of this same set of data (see [1]) has also relied on basic VAR analysis, there had been some common shortcomings to the validity and scope of results, which we were able to remedy here, thus solving statistical shortcomings while also presenting completely new results in a clearer more clinically oriented fashion. How we were able to achieve this, the presentation of a newly developed optimized multivariate lag selection process in VAR analysis, and a comprehensive review of the principles of vector autoregression will be presented next.

### 2.2. Reviewing Vector Autoregression as a Quantitative Approach to Time Series Data

The use of vector autoregressive models (VAR) for the analysis of time series data in psychosomatic medicine (also widely used in neuroscience) allows treating a set of variables as jointly driven by the lagged values of all variables in the system with no a priori assignment of dependent and independent status being necessary. This technique seems particularly apt for research in psychosomatic medicine, where [21], among others, has long called for a more integrated (monistic) view on the complexity of dynamic dependencies and intertemporal reciprocal cause and effect relationships among different psychic as well as somatic variables.

Any VAR model requires the user to select a maximum number of lags, which, in more practical terms, refers to how far back in time the user wants to go in the search for past recordings of all variables to predict the present value of one variable. The farther back in time the user decides to go, the more explanatory variables (lags) need to be included in the model because it used to be improper to exclude past recordings of explanatory variables, which lay in-between the present value and the most historic one [22, 23].

Unfortunately including more explanatory variables (going back further in time) is a double edged sword, since this would provide a VAR model more representative of reality (goodness of fit), but would also endorse one with less explanatory power (lower adjusted *R* ^2^). The latter is due to the tremendous penalty inflicted by the large number of explanatory variables (lags) in the model resulting in high estimation variance [22, 23]. This substantial drawback weakened the substance of empirical findings derived from VAR models, because researchers would either present results through models with teeth chattering low *R* ^2^ values (see previously published results from the same raw data as one example) or adopt models only incorporating the effects of events preceding the predicted value of a variable by one day/one unit of time in the VAR (e.g., see [24]).

In order to alleviate this shortcoming of low adjusted *R* ^2^ values in the standard vector autoregressive modeling approach, we developed a computer code implementing a statistical procedure recently published in parts in Savin and Winker [25] and Winker [26, 27], referred to as the optimized multivariate lag selection process, which allows (contrary to previous practice) excluding such explanatory variables (lags) from the VAR model which add little to its goodness of fit (estimated representativeness of reality) while nonetheless reducing its explanatory power (adjusted *R* ^2^). This “admittance of holes” to the lag structure (equations organizing the explanatory variables) allows us to now present an entirely new model exhibiting more detailed dynamics with a smaller number of parameters, for the data in this case resulting in about tenfold increase of the adjusted *R* ^2^ value. Mathematical details of applying the optimized multivariate lag selection process to this VAR analysis of the ten time series of the data set at hand will be presented next (and may be skipped by the more clinically focused researcher).

### 2.3. Applying the Optimized Multivariate Lag Selection Process

A standard vector autoregressive (VAR) model was constructed, using EViews 7.1 (QMS, Quantitative Micro Software, Irvine CA), based on the ten time series we mentioned above. In order to focus on the innovative aspects of our methodology we will not delve into the details of VAR model construction, which have been described at length in preceding publications (i.e., [1, 24]).

Given the large number of explanatory variables (the more lags, the more variables) and the limited number of observations, only a very limited number of lags (past days) could be considered while adjusted *R* ^2^ would still be low, if we were to follow the standard modeling approach [22, 23]. The novel contribution is to maximize the informational content of the model by minimizing an information criterion [25–27].

In more concrete terms, if we assume that any one value within the ten time series may have effects on any of the other values of all-time series with a delay of up to one week, a total of 710 parameters would have to be estimated. Given 120 observations in each time series, this results in tremendous estimation variance (very low *R* ^2^). Model selection criteria suggest using only one lag (assuming effects will take place within a day instead of within a week, which seems highly unrealistic but is a common approach adopted by other researchers in the field, including Wild et al., 2010) resulting in a total of only 110 parameters to be estimated with a still low *R* ^2^ value of 0.02 for the model explaining glycemic variance [1].

To resolve this dilemma, we drew on Winker [26, 27] and Savin and Winker [25] engaging in optimized multivariate lag structure analysis. Given the huge discrete search space of all possible lag structures, for example, for a maximum lag length of seven, heuristic optimization algorithms are used to this end. For this process, a computer code was developed using Matlab R2011b with an interface to EViews 7.1, which implements a Genetic Algorithm for the search of an optimized lag structure making use of information criteria (BIC) as in the standard selection procedure (see for more details [25]). By providing an approximation to the minimum of the information criterion, the resulting model exhibits an optimized tradeoff between a good fit to the multivariate dynamics of the data and model parsimony.

As a result, we obtained a model with only 70 parameters, but still cover effect delays up to one week. Since the maintained lags are selected based on their joint informational content (as measured by the information criteria), the procedure results in a model with much higher explanatory power (for predicting glycemic variability adjusted *R* ^2^ value of 0.20 as opposed to 0.02 for the standard model with only one lag) and a richer dynamic.

Given the rich dynamics between all variables of the model, besides considering single equations, the calculation of impulse response functions as in [1] would be of interest. However, the zero constraints of the VAR model with holes preclude the application of standard methods for the calculation of confidence bands.

Similarly, poor glycemic control (high glycemic variability) will correlate with low glycemic variability four days earlier, a calm mother three days earlier, an excited mother seven days earlier, a dominating mother four days earlier, a nondominating mother seven days earlier (although statistically insignificant), a sad father both five and six days earlier, a calm father both three and seven days earlier, and a dominating father both two and five days earlier. High glycemic variability will also correlate with a sad child six days later, an excited mother three days later, and a dominating father one day later. For a graphical representation see Figure 2.

## 3. Results and Discussion

The optimized multivariate lag structure selection process provides one equation of seemingly unrelated multiple regression for each of the ten time series to be presented next. Three of them directly involve glycemic variability in addition to the one for glycemic variability itself, which shall be presented last (lags in parentheses):

 affective valence of the adolescent = *α*_1_ glycemic variability (−6) + *α*_2_ valence adolescent (−1) (*R* ^2^ = 0.25, adj. *R* ^2^ = 0.24);
 affective valence of the mother = *α*_3_ dominance adolescent (−7) + *α*_4_ valence mother (−5) + *α*_5_ arousal mother (−6) + *α*_6_ arousal father (−4) + *α*_7_ arousal father (−6) (*R* ^2^ = 0.21, adj. *R* ^2^ = 0.18);
 affective valence of the father = *α*_8_ valence adolescent (−3) + *α*_9_ valence adolescent (−5) + *α*_10_ arousal mother (−5) + *α*_11_ dominance father (−3) (*R* ^2^ = 0.21, adj. *R* ^2^ = 0.18);
 arousal of the adolescent = *α*_12_ arousal adolescent (−1) + *α*_13_ arousal adolescent (−3) + *α*_14_ arousal adolescent (−7) + *α*_15_ valence mother (−4) + *α*_16_ arousal mother (−3) + *α*_17_ valence father (−2) + *α*_18_ valence father (−6) (*R* ^2^ = 0.30, adj. *R* ^2^ = 0.25);
 arousal of the mother = *α*_19_ glycemic variability (−3) + *α*_20_ arousal adolescent (−7) + *α*_21_ dominance adolescent (−5) + *α*_22_ arousal mother (−5) + *α*_23_ arousal mother (−7) + *α*_24_ dominance mother (−1) + *α*_25_ dominance father (−6) (*R* ^2^ = 0.29, adj.* R* ^2^ = 0.24);
 arousal of the father = *α*_26_ valence mother (−4) + *α*_27_ dominance mother (−6) + *α*_28_ arousal father (−1) + *α*_29_ arousal father (−2) + *α*_30_ arousal father (−6) + *α*_31_ dominance father (−1) (*R* ^2^ = 0.19, adj. *R* ^2^ = 0.15);
 dominance of the adolescent = *α*_32_ valence adolescent (−1) + *α*_33_ arousal adolescent (−5) + *α*_34_ arousal father (−1) + *α*_35_ dominance father (−1) (*R* ^2^ = 0.25, adj. *R* ^2^ = 0.22);
 dominance of the mother = *α*_36_ valence mother (−7) + *α*_37_ dominance mother (−1) + *α*_38_ dominance mother (−3) + *α*_39_ dominance father (−5) (*R* ^2^ = 0.65, adj. *R* ^2^ = 0.64);
 dominance of the father = *α*_40_ glycemic variability (−1) + *α*_41_ dominance child (−6) + *α*_42_ valence mother (−5) + *α*_43_ valence mother (−7) + *α*_44_ dominance mother (−4) + *α*_45_ dominance mother (−6) + *α*_46_ valence father (−1) + *α*_47_ valence father (−3) + *α*_48_ arousal father (−3) + *α*_49_ dominance father (−2) (*R* ^2^ = 0.34, adj.* R* ^2^ = 0.27);
 
*glycemic variability* = ß_1_ glycemic variability (−4) + ß_2_ arousal mother (−3) + ß_3_ arousal mother (−7) + ß_4_ dominance mother (−4) + ß_5_ dominance mother (−7) + ß_6_ valence father (−5) + ß_7_ valence father (−6) + ß_8_ arousal father (−3) + ß_9_ arousal father (−7) + ß_10_ dominance father (−2) + ß_11_ dominance father (−5) (*R* ^2^ = 0.28, adj. *R* ^2^ = 0.20).

The coefficients, their standard error, *t*-statistic, and probability referred to above, can be reviewed in Table 1.

The development of a novel statistical methodology allowed us to disentangle the data and generate statistically reliable results in the form of ten equations. The dynamic of the results pertaining to glycemic variability, (thereby, it has to be taken into account that additional dynamic interactions arise due to spillover between equations, which are not considered here), taking into account the direction of coefficients, can be summarized in the following words and graphical representations.

**Figure 1:**
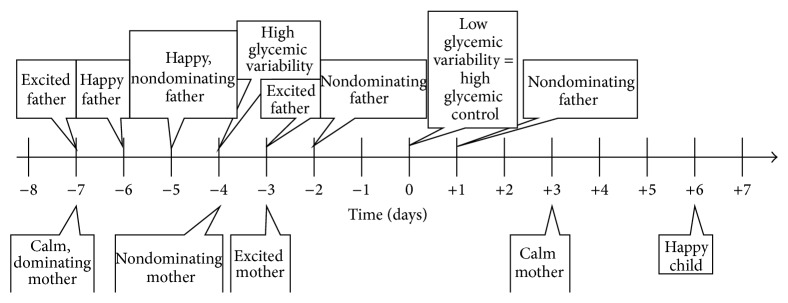
Timeline displaying effects correlating with high glycemic control. The graph depicts a psychosomatic cycle in which various emotional states of all involved family members influence glycemic variability of the adolescent patient and vice versa.

*Low* glycemic variability and, therefore,* good* diabetic control will correlate with the following: high glycemic variability four days earlier, an excited mother three days earlier, a calm mother seven days earlier, a non-dominating mother four days earlier, a dominating mother seven days earlier (although statistically insignificant), a happy father both five and six days earlier, an excited father both three and seven days earlier, and a non-dominating father both two and five days earlier. Low glycemic variability will also correlate with a happy child six days later, a calm mother three days later, and a non-dominating father one day later. For a graphical representation of this paragraph refer to Figure 1.

**Figure 2:**
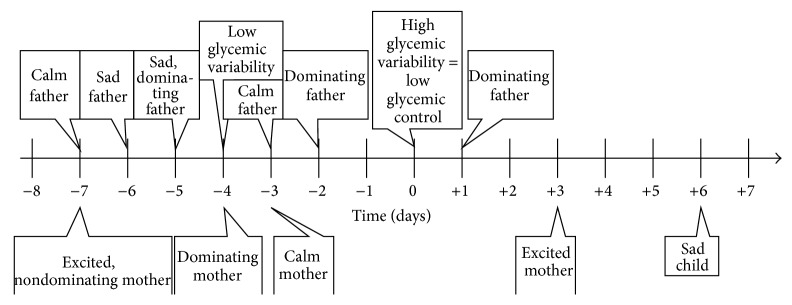
Timeline displaying effects correlating with poor glycemic control. The graph depicts a psychosomatic cycle in which various emotional states of all involved family members influence glycemic variability of the adolescent patient and vice versa.

Similarly, poor glycemic control (high glycemic variability) will correlate with low glycemic variability four days earlier, a calm mother three days earlier, an excited mother seven days earlier, a dominating mother four days earlier, a non-dominating mother seven days earlier (although statistically insignificant), a sad father both five and six days earlier, a calm father both three and seven days earlier, and a dominating father both two and five days earlier. High glycemic variability will also correlate with a sad child six days later, an excited mother three days later, and a dominating father one day later. A graphical representation of this paragraph is presented in Figure 2

In clinical terms, this means, good diabetic control was preceded by attentive and alert (“high arousal,” excited) parents with a positive attitude (“happy father”), at the same time refraining from too much overwhelming presence (“low dominance”). Likewise, phases of good diabetic management were followed by a continuously distant father (“low dominance”), unfortunately a less alert mother (“low arousal”), and a content (“happy”) adolescent index patient.

**Figure 3:**
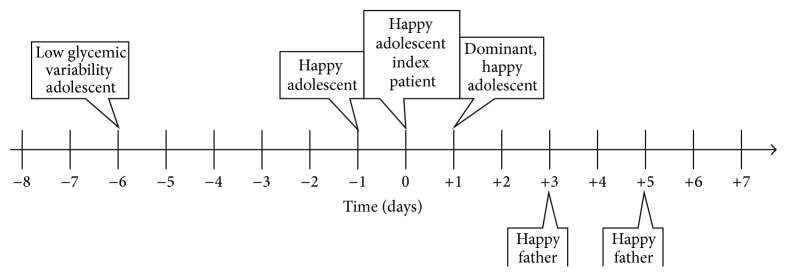
Timeline displaying effects correlating with affective valence in the adolescent index patient. The graph depicts a psychosomatic cycle in which various emotional states of all involved family members influence affective valence (pleasure) of the adolescent patient and vice versa.

**Figure 4:**
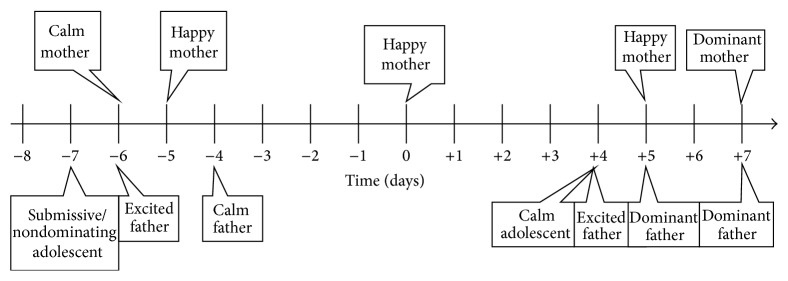
Timeline displaying effects correlating with affective valence in the mother of the adolescent index patient. The graph depicts a psychosomatic cycle in which various emotional states of all involved family members influence affective valence (pleasure) of the mother to the adolescent patient and vice versa.

**Figure 5:**
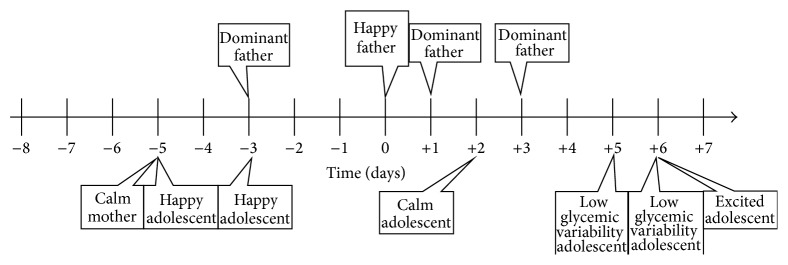
Timeline displaying effects correlating with affective valence in the father of the adolescent index patient. The graph depicts a psychosomatic cycle in which various emotional states of all involved family members influence affective valence (pleasure) of the father to the adolescent patient and vice versa.

Similarly, mostly self-explanatory, graphical representations were constructed for the effects surrounding the affective valence of all three family members (see Figures 3, 4, and 5). We picked these three timelines for more detailed examination, because the appropriate measurement of depressive symptoms (which at least at a distance somewhat relates to affective valence) in diabetics in general, remains to be a topic of current debate in the literature [28].

## 4. Conclusions

In comparison to the results derived from the same set of raw data with a different statistical approach in an earlier publication [1], there are several improvements we were able to achieve:

(i) increasing the coefficient of determination *R* ^2^ for the model prediction of glycemic variability by factor ten (adjusted *R* ^2^ value of 0.20 as opposed to 0.02) while incorporating significant effects of explanatory variables (lags) stemming from a longer period of time preceding the predicted event;
(ii) presenting a more precise timeline of effects of various variables on each other, including glycemic variability and vice versa (e.g., “a nondominating mother four days prior to a set day will increase glycemic control” instead of “a nondominating mother somewhere up to four days prior to a set day will increase glycemic control”);
(iii) isolating additional relationships between variables which did not reach statistical significance earlier or took more time to take effect than the time frame of the earlier models allowed for.

A more substantial contribution of this paper is the demonstration and practical application of the multivariate lag selection process to VAR analysis, resolving an essential shortcoming in VAR analysis of (relatively) small samples. Hence, this contribution to literature will have relevance beyond the case study approach but also to VAR-based studies of larger cohorts of patients (as e.g., [24]), significantly increasing either the number of effects analyzed (as in [24]) or the statistical reliability (i.e., the adjusted *R* ^2^) with which results are presented.

All in all, however, mathematically refined quantitative methodological approaches relying on modern computational technology can generate more specific, reproducible, and thus trustworthy results than purely qualitative (narrative) accounts, while still honoring the benefits of the case study approach aiming to explore previously unforeseen avenues fit for further vested inquiry (often costly to perform).

Yet, we have to ask ourselves critically if the added mathematical complexity honors the overall value of the results a case study approach can provide. Revisiting the opening comments of this report in the context of brittle diabetes, it seems interesting to note that particularly the most highly acclaimed and clinically widely trusted research on brittle diabetes has also been the most severely and broadly criticized. So, for instance, more than ten years after the initial publication of the pioneering work of Minuchin et al. in 1978 (on what they called “psychosomatic diabetes”) entitled “Psychosomatic families” [2], critics commented as follows: “…as we conducted research and therapy with the families of diabetic children, we were impressed with both the limit of the formulation of the family's role in diabetes offered in ‘Psychosomatic Families' and the uncritical acceptance that the book continued to enjoy” [29]. In their rather pointed article entitled “The ‘psychosomatic family' reconsidered II: recalling a defective model and looking ahead” Coyne and Anderson [29] criticize Minuchin et al. [2] primarily for their bold, yet statistically (allegedly) poorly supported, statements on the “typical psychosomatic family” (Minuchin et al. [2] describe the “psychosomatic family” as featuring enmeshment, rigidity, overprotectiveness, and lack of conflict resolution and the children affected by brittle diabetes as having difficulty in handling stress, showing a tendency to internalize anger and being somewhat immature in their ability to cope with challenging situations) and their overgeneralizations of these overall “weak” findings on familial situations in one psychosomatic illness to various psychosomatic illnesses. More specifically, small sample sizes and poor documentation of methodology (or lack thereof) are being highlighted.

Reflecting on such valid criticism in light of our own extensive research both on the subject of brittle diabetes in adolescents and on the various shortcomings of contemporary statistical approaches to time series data in psychosomatic medicine, we believe there is a case for both sides. On the one hand, we must vigorously support critics (i.e., [29]) in their call for much more detailed and sophisticated reports on and publication of statistical methodology in such complex and intricate research situations as are present in multivariate time series analysis. The reason lies in the fact that there is vast room for pitfalls and error with this type of research, if left in the hands of the mathematically inexperienced. On the other hand, however, we found for fact, that with the change of statistical approach, the results drawn from a given set of data may change somewhat, despite both methodologies being perfectly valid and academically accepted. So one wonders how this (agreeably small) imprecision of highly quantitative research is any different from the (possibly but not necessarily larger) inaccuracy of qualitative research due to subjectivity. Noteworthy, and in taking up the cudgels for Minuchin et al. [2, 11], the one finding which we were able to observe clinically before conducting any statistical testing at all, namely, that of a dominating mother having a negative effect on glycemic control of her child, was also a finding that both of our methodologies were able to report at a high level of significance. (Amusingly, one might find what Minuchin et al. [2] described as overprotectiveness in families with brittle diabetes is very similar, if not the same, to what we were able to pinpoint in terms of exaggerated control of a mother over her glycemically out of control child.) Additionally, we also fear that critics of primarily qualitative case research (i.e., [2]) may not have realized the vastness of data inherent even in a small sample in time series analysis, an apprehension possibly supported by the fait accompli of not too many critics providing any statistically evidenced findings on the subject of brittle diabetes themselves (i.e., [29]). So in conclusion, we believe the careful observation of the clinically experienced therapist to be almost as valuable as the most substantiated and savvy statistical approach.

## Figures and Tables

**(a):** 

	Coefficient	Std. error	*t*-statistic	Prob.
*α*_1_	0.008371	0.002505	3.341682	0.0009
*α*_2_	0.439050	0.071648	6.127902	0.0000
*α*_3_	0.196661	0.072361	2.717768	0.0067
*α*_4_	0.193472	0.070105	2.759765	0.0059
*α*_5_	0.166062	0.072169	2.301002	0.0216
*α*_6_	−0.093081	0.038780	−2.400229	0.0166
*α*_7_	0.083885	0.023675	3.543200	0.0004
*α*_8_	−0.133217	0.045307	−2.940347	0.0033
*α*_9_	0.135556	0.044104	3.073571	0.0022
*α*_10_	−0.096673	0.029864	−3.237170	0.0012
*α*_11_	−0.220601	0.061646	−3.578496	0.0004
*α*_12_	−0.083390	0.031821	−2.620595	0.0089
*α*_13_	0.167024	0.043985	3.797288	0.0002
*α*_14_	0.499978	0.148744	3.361336	0.0008
*α*_15_	0.235265	0.063599	3.699206	0.0002
*α*_16_	−0.118392	0.039810	−2.973946	0.0030
*α*_17_	−0.177384	0.058985	−3.007251	0.0027
*α*_18_	0.327619	0.062900	5.208601	0.0000
*α*_19_	−0.006755	0.002888	−2.339111	0.0195
*α*_20_	−0.516945	0.178245	−2.900191	0.0038
*α*_21_	−0.973039	0.242951	−4.005083	0.0001
*α*_22_	0.190612	0.063265	3.012915	0.0026
*α*_23_	−0.212629	0.060467	−3.516477	0.0005
*α*_24_	−0.560562	0.136662	−4.101828	0.0000
*α*_25_	−0.464339	0.146477	−3.170045	0.0016
*α*_26_	−0.090665	0.041861	−2.165871	0.0305
*α*_27_	0.447149	0.069911	6.395994	0.0000
*α*_28_	0.234203	0.065907	3.553560	0.0004
*α*_29_	−0.225144	0.058588	−3.842809	0.0001
*α*_30_	0.129774	0.038175	3.399442	0.0007
*α*_31_	0.182089	0.037975	4.795004	0.0000
*α*_32_	−0.077998	0.029281	−2.663826	0.0078
*α*_33_	−0.325788	0.065003	−5.011909	0.0000
*α*_34_	0.215753	0.065266	3.305758	0.0010
*α*_35_	−0.259613	0.081614	−3.181004	0.0015
*α*_36_	0.200644	0.061428	3.266334	0.0011
*α*_37_	0.292372	0.060802	4.808558	0.0000
*α*_38_	−0.186054	0.064022	−2.906069	0.0037
*α*_39_	−0.233369	0.086570	−2.695740	0.0071
*α*_40_	0.004900	0.001217	4.024947	0.0001
*α*_41_	0.367140	0.102177	3.593182	0.0003
*α*_42_	−0.128680	0.045575	−2.823477	0.0048
*α*_43_	−0.111369	0.043503	−2.560006	0.0106
*α*_44_	−0.186954	0.067466	−2.771067	0.0057
*α*_45_	−0.187772	0.065392	−2.871465	0.0042
*α*_46_	−0.192931	0.048915	−3.944164	0.0001
*α*_47_	−0.201673	0.062378	−3.233079	0.0013
*α*_48_	−0.092639	0.048991	−1.890956	0.0589
*α*_49_	0.154373	0.062922	2.453387	0.0143

Determinant residual covariance 9.14*E* − 05.

**(b):** 

	Coefficient	Std. error	*t*-statistic	Prob.
*β*_1_	−0.197322	0.076111	−2.592545	0.0097
*β*_2_	3.639513	1.583793	2.297973	0.0218
*β*_3_	−4.889116	1.647518	−2.967565	0.0031
*β*_4_	22.52994	3.969363	5.675959	0.0000
*β*_5_	−6.340918	3.554736	−1.783794	0.0747
*β*_6_	9.565170	3.704850	2.581797	0.0100
*β*_7_	9.249940	2.865721	3.227788	0.0013
*β*_8_	7.562806	2.651011	2.852801	0.0044
*β*_9_	10.96846	2.600148	4.218400	0.0000
*β*_10_	13.04606	3.522259	3.703891	0.0002
*β*_11_	11.03846	4.583850	2.408120	0.0162

Determinant residual covariance  9.14*E* − 05.

## Appendix

See Table 1.

## Abbreviations

VAR: vector autoregression/vector autoregressive.
